# Oxidation of Cyclohexane to Cyclohexanol/Cyclohexanone Using Sol‐Gel‐Encapsulated Unspecific Peroxygenase from *Agrocybe aegerita*

**DOI:** 10.1002/open.202400152

**Published:** 2024-08-30

**Authors:** Yinqi Wu, Frank Hollmann, Musa M. Musa

**Affiliations:** ^1^ Department of Biotechnology Delft University of Technology 2629HZ Delft The Netherlands; ^2^ Department of Chemistry Interdisciplinary Research Center for Refining and Advanced Chemicals King Fahd University of Petroleum and Minerals Dhahran 31261 Saudi Arabia

**Keywords:** Biocatalysis, Unspecific peroxygenase, Oxyfunctionalisation, Cyclohexane, sol-gel encapsulation

## Abstract

Unspecific peroxygenase from *Agrocybe aegerite* (*Aae*UPO) is a remarkable catalyst for the oxyfunctionalization of non‐activated C−H bonds under mild conditions. It exhibits comparable activity to P450 monooxygenase but offers the advantage of using H_2_O_2_ instead of a complex electron transport chain to reductively activate O_2_. Here, we demonstrate the successful oxidation of cyclohexane to cyclohexanol/cyclohexanone (KA‐oil) using sol‐gel encapsulated *Aae*UPO. Remarkably, cyclohexane serves both as a solvent and a substrate in this system, which simplifies product isolation. The ratio of cyclohexanone to cyclohexanol using this approach is remarkably higher compared to the oxidation using free *Aae*UPO in aqueous media using acetonitrile as a cosolvent. The utilization of sol‐gel encapsulated *Aae*UPO offers a promising approach for oxyfunctionalization reactions and improves the chances for this enzyme to be incorporated in the same pot with other chemical transformations.

## Graphical abstract

Sol‐gel immobilisation efficiently protects a peroxygenase (*Aae*UPO) from inactivation through contact with the hydrophobic organic phase (cyclohexane). A promising catalyst preparation to synthesise KA‐oil is presented.

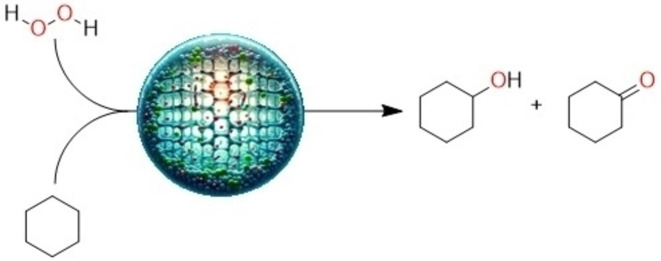

## 1 Introduction

The oxidation of cyclohexane to cyclohexanol/cyclohexanone [known as ketone‐alcohol oil (KA‐oil)] is an industrially important reaction as it is the main route for the production of ϵ‐caprolactone, ϵ‐caprolactam, and adipic acid, which are heavily used in the polymer industry as feed stocks for nylon 6 and nylon 6,6.[^1^, ^2^] Chemical routes for the transformation of cyclohexane to KA‐oil demand relatively harsh conditions of temperature and/or pressure,[^3^, ^4^] thereby increasing the operational expenses. Furthermore, to avoid undesired overoxidation, cyclohexanol/one concentration must be maintained low resulting in overall low conversion.

Recently, unspecific peroxygenases (UPOs), particularly the UPO from *Agrocybe aegerita* (*Aae*UPO, EC 1.11.2.1)[^5^, ^6^, ^7^, ^8^] have emerged as promising catalysts for selective oxyfunctionalisation reactions.[^9^, ^10^, ^11^] Earlier studies have demonstrated *Aae*UPO's potential for selective (i. e., yielding cyclohexanol and cyclohexanone only) oxidation of cycloalkanes.[^12^, ^13^, ^14^] Using monophasic reaction systems in the presence of 50 % (v/v) acetonitrile to solubilise higher concentrations of cyclohexane resulted in production of more than 300 mM of KA‐oil with a productivity of 157 mM h^−1^.^[15]^ Such reaction setups, however, do not bear great promise for preparative applications due to issues in the downstream processing [(DSP), caused e. g. by azeotropes]. Therefore, we hypothesised that so‐called two liquid phase systems may represent a doable approach to attain high product titres while maintaining simplicity in DSP. Initial experiments using soluble *Aae*UPO in the presence of various water‐immiscible solvents revealed a certain incompatibility resulting in irreversible *Aae*UPO denaturation, presumably at the liquid‐liquid interphase.

Enzyme encapsulation is a promising strategy for enhancing enzyme performance and expanding their utility in organic synthesis.[^16^, ^17^, ^18^, ^19^, ^20^] This technique plays a vital role in enabling the effective utilisation of enzymes on large scales for the production of fine chemicals by enhancing enzyme activity, stability, and reusability. Among the established methods for encapsulation is the utilisation of a sol‐gel matrix to encapsulate enzymes.[^21^, ^22^]

**Scheme 1:**
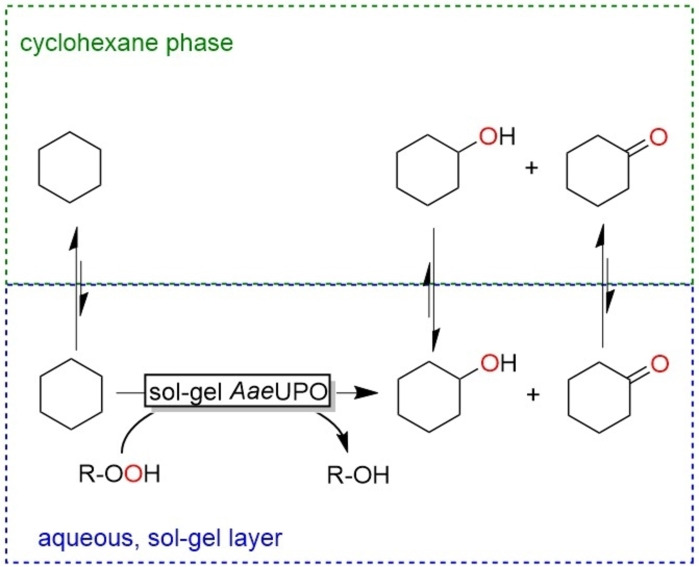
Oxyfunctionalisation of cyclohexane using sol‐gel‐encapsulated AaeUPO in a biphasic reaction system. Note, *Aae*UPO catalyses both, the hydroxylation of cyclohexane and of cyclohexanol (yielding a gem‐diol spontaneously eliminating water to produce cyclohexanone).

We reasoned that sol‐gel encapsulation of *Aae*UPO may alleviate the previously observed inactivation by avoiding a direct contact between the enzyme and the interphase. Overall, a biphasic reaction scheme comprising cyclohexane as substrate reservoir and product sink for the *Aae*UPO‐catalysed hydroxylation of cyclohexane was envisioned (Scheme 1).

## Experimental Procedures

### General

All chemicals utilised in the experiments were obtained from commercial suppliers and used without additional processing. H_2_O_2_ was acquired as a 50 % (w/w) solution in water, while *t*BuOOH was procured as a 5.0 M solution in decane. Phosphate buffer solutions were adjusted to pH 6.0 at room temperature. Gas chromatography analyses were conducted using a Shimadzu GC‐2010 plus with FID equipped with an Agilent CP‐Wax 52GB column (50 m ×0.53 mm ×2.0 μm), and using N_2_ as the carrier gas.

### *Aae*UPO

The expression and purification of *Aae*UPO (engineered variant, commonly known as PaDa−I)^[8]^ were previously carried out in a 2500‐L pilot‐scale cultivation of recombinant *Pichia pastoris* X‐33.^[23]^ Enzyme stock solutions at a concentration of 56 μM were stored in phosphate buffer (100 mM, pH 8.0) at −28 °C.

### Sol‐Gel Encapsulation of *Aae*UPO

The sol‐gel was prepared following a previously published procedure.^[24]^ Briefly, the silica sol was prepared by combining tetramethyl orthosilicate (TMOS, 4.20 g), distilled water (0.94 g) and HCl (30 μL of 100 mM HCl solution), and mixing the solution until a single layer formed. Gels were prepared by mixing equal volumes of the above sol solution and the enzyme stock solution in an Eppendorf tube. The enzyme stock was in phosphate buffer (100 mM, pH 8) containing 56 μM *Aae*UPO. The sol‐gel was then incubated in the fridge for 24 h for gel aging. This hydrogel was dried at RT in air for 12 h to give hydrated silica SiO_2_.nH_2_O, the so‐called xerogel. The xerogel was then extensively washed with phosphate buffer (100 mM, pH 6.0) to remove any remaining methanol.

### Oxidation of Cyclohexane using Xerogel‐Encapsulated *Aae*UPO

To the xerogel‐encapsulated *Aae*UPO [prepared by mixing sol solution (2.0 mL) and of *Aae*UPO solution (2.0 mL)] was added cyclohexane (4.0 mL) in a 15 mL glass reaction vial. The reaction was initiated by the addition of H_2_O_2_ or *t*BuOOH at a rate of 10 mM h^−1^. The reaction mixture was stirred at 25 °C at 900 rpm. Samples (50 μL) were withdrawn from the reaction mixture and diluted with 450 μL of ethyl acetate containing dodecane (5.0 mM) as internal standard then analysed by GC.

### GC Analysis

The following temperature gradient was used for all GC analyses: (Split 10), 90 °C hold for 3 min, 10 °C/min to 180 °C hold 1 min, 30 °C/min to 230 °C hold 1 min. The concentrations of cyclohexanol and cyclohexanone were calculated using calibration curves developed before.^[15]^

## 2 Results and Discussion

**Figure 1:**
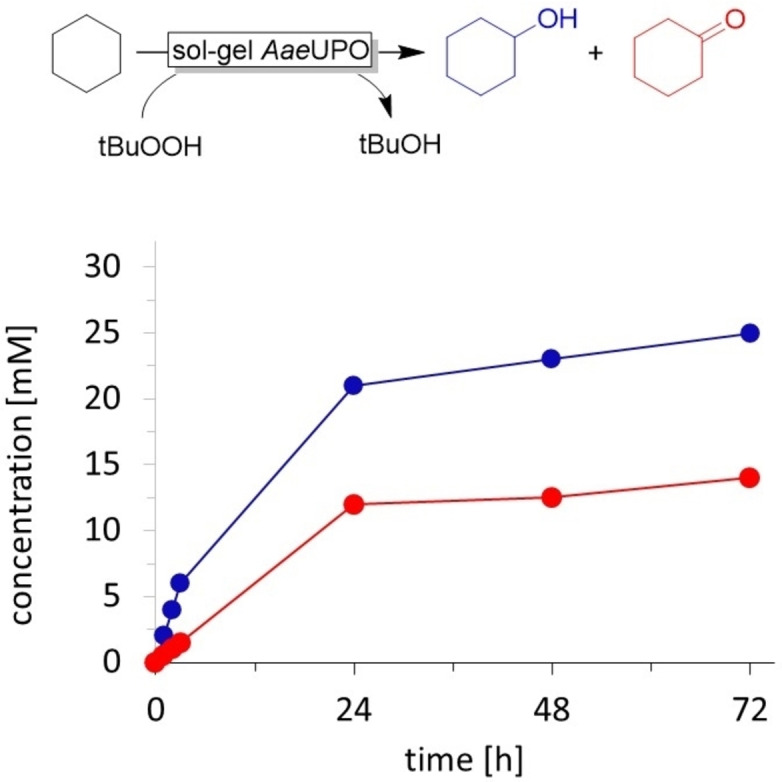
Reaction progress of cyclohexane oxidation to cyclohexanol (•) and cyclohexanone (•) using xerogel‐encapsulated *Aae*UPO and *t*BuOOH as oxidant. The reaction conditions included 4 mL of cyclohexane, 2 mL of 56.5 μM enzyme solution encapsulated using the sol‐gel method, at 25 °C and 900 rpm. *t*BuOOH dosing rate of 10 mM h^−1^. n=1

To estimate the catalytic activity of the sol‐gel encapsulated *Aae*UPO, we used it in a biphasic reaction system. We suspended the sol‐gel in pure cyclohexane at ambient temperature and continuously added *t*BuOOH at a feed rate of 10 mM h^−1^ (Figure 1).

The ‘initial’ product formation rate (24 h) was approximately 0.83 mM h^−1^ corresponding to a *Aae*UPO turnover frequency of roughly 0.5 min^−1^. This corresponds to a formal utilisation of only 8.3 % of the *t*BuOOH added. Possibly diffusion limitation and/or unfavourable partitioning of the oxidant between the aqueous and organic layer may account for this. Using H_2_O_2_ as stoichiometric oxidant resulted in significantly higher conversion rates (*vide infra*). It is also worth mentioning that in these experiments already from an early stage onwards conversion of cyclohexanol to cyclohexanone was observed. In previous experiments using soluble *Aae*UPO in aqueous media, this was observed only once the initial cyclohexane substrate had been mostly consumed.[^12^, ^13^, ^14^] We attribute this difference to the biphasic nature of the reaction system where the more polar cyclohexanol partially accumulates in the aqueous phase and thereby dominates over the less polar cyclohexane.

**Figure 2:**
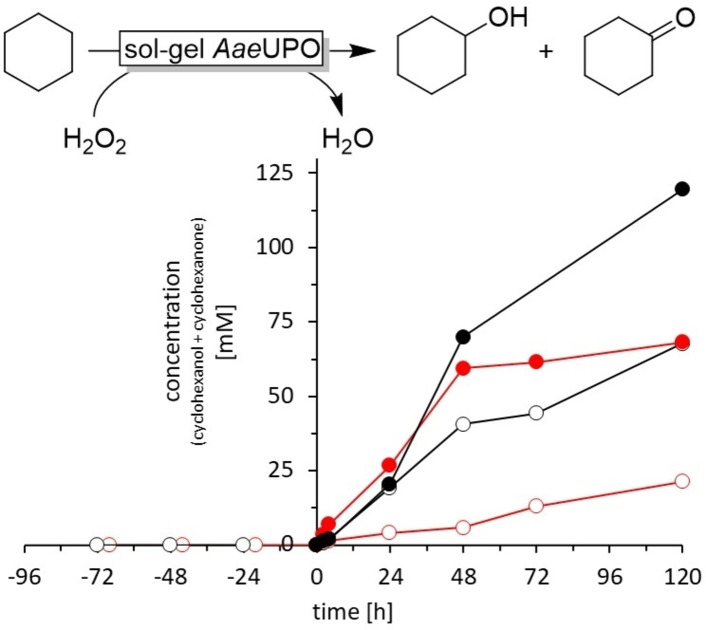
Performance of the sol‐gel immobilised *Aae*UPO in cyclohexane using H_2_O_2_ as stoichiometric oxidant. (•) sol‐gel immobilised *Aae*UPO, and (•) soluble *Aae*UPO with immediate H_2_O_2_ dosage; (○) sol‐gel immobilised *Aae*UPO, and (○) soluble *Aae*UPO preincubated in the reaction mixture for 72 h prior starting the reaction by initiating the H_2_O_2_ dosage. The reaction conditions included 3.75 mL of cyclohexane, 250 μL of acetonitrile, 2.0 mL of 56.5 μM enzyme solution encapsulated using the sol‐gel method, at 25 °C and 900 rpm. H_2_O_2_ dosing rate of 10 mM h^−1^. n=1

Next, we evaluated aqueous H_2_O_2_ as stoichiometric oxidant (Figure 2). Performing the transformation under the same conditions, as shown in Figure 1, but substituting *t*BuOOH by H_2_O_2_, a significant rate acceleration was observed (Figure 2a). The initial product formation rate increased to 1.25 mM h^−1^ and after 72 h more than twice as much product had been formed. Interestingly, the cyclohexanol to cyclohexanone ratio was close to equimolar.

We next compared the stability of free and immobilised *Aae*UPO in the presence of cyclohexane (Figure 2). Free *Aae*UPO lost its activity already after 48 h reaction time (Figure 2, •). To validate that this apparent inactivation could be attributed to the interaction with the organic phase, we performed a preincubation experiment in which the enzyme was exposed to the reaction conditions albeit without addition of H_2_O_2_. After 72 h, the transformation was initiated revealing a more than 80 % reduction of the initial product formation rate (Figure 2, ○). Repeating the same experiments with sol‐gel immobilised *Aae*UPO (Figure 2, • and •) revealed a much less pronounced difference between pre‐incubated and freshly used enzyme; in both cases the reaction proceeded for at least 120 h. We interpret these observations by a stabilising effect of the sol‐gel encapsulation on the structural integrity of *Aae*UPO.

The ability of sol‐gel encapsulated *Aae*UPO to perform oxidation of cyclohexane in non‐aqueous media presents an exciting prospect for incorporating this reaction with other interesting transformations. One particularly promising avenue is the potential to couple the *Aae*UPO‐catalysed oxidation of cyclohexane with the formation of ϵ‐caprolactone from cyclohexanone in one pot.

## 3 Conclusions

In this study, we have established sol‐gel immobilisation of an unspecific peroxygenase for the application in two liquid phase systems. The encapsulated enzyme exhibited significant catalytic activity under these conditions. Presumably phase transfer limitations currently limit the overall performance of the system, which will be investigated in greater detail in future studies.

## Conflict of Interests

The authors declare no conflict of interest.

4

## Data Availability

The data that support the findings of this study are available from the corresponding author upon reasonable request.
